# Digital Health Screening in People With HIV in Uganda to Increase Alcohol Use Reporting: Qualitative Study on the Development and Testing of the Self-administered Digital Screener for Health

**DOI:** 10.2196/35015

**Published:** 2022-09-01

**Authors:** Nneka Emenyonu, Allen Kekibiina, Sarah Woolf-King, Catherine Kyampire, Robin Fatch, Carol Dawson-Rose, Winnie Muyindike, Judith Hahn

**Affiliations:** 1 Division of HIV, Infectious Diseases, and Global Medicine Department of Medicine University of California San Francisco San Francisco, CA United States; 2 MUST Grants Office Mbarara University of Science and Technology Mbarara Uganda; 3 Department of Psychology Syracuse University Syracuse, NY United States

**Keywords:** unhealthy alcohol use, HIV, digital screening, Uganda, mobile phone

## Abstract

**Background:**

Alcohol consumption is a critical driver of the HIV epidemic worldwide, particularly in sub-Saharan Africa, where unhealthy alcohol use and HIV are prevalent. Brief alcohol interventions are effective in reducing alcohol use; however, they depend on effective screening for unhealthy alcohol use, which is often underreported. Thus, there is a need to develop methods to improve reporting of unhealthy alcohol use as an essential step toward referral to brief alcohol interventions. Self-administered digital health screeners may improve reporting.

**Objective:**

This study aimed to develop and test a digital, easy-to-use self-administered health screener. The health screener was designed to be implemented in a busy, underresourced HIV treatment setting and used by patients with varying levels of literacy.

**Methods:**

We conducted a qualitative study at the Immune Suppression Syndrome (ISS) Clinic of Mbarara Regional Referral Hospital in Uganda to develop and test a digital self-administered health screener. The health screener included a training module and assessed behaviors regarding general health, HIV care, and mental health as well as sensitive topics such as alcohol use and sexual health. We conducted focus group discussions with clinicians and patients with HIV of the Mbarara ISS Clinic who consumed alcohol to obtain input on the need for and content, format, and feasibility of the proposed screener. We iteratively revised a tablet-based screener with a subset of these participants, piloted the revised screener, and conducted individual semistructured in-depth interviews with 20 participants who had taken part in our previous studies on alcohol and HIV, including those who had previously underreported alcohol use and with low literacy.

**Results:**

A total of 45 people (n=5, 11% clinicians and n=40, 89% Mbarara ISS Clinic patients) participated in the study. Of the patient participants, 65% (26/40) were male, 43% (17/40) had low literacy, and all (40/40, 100%) had self-reported alcohol use in previous studies. Clinicians and patients cited benefits such as time savings, easing of staff burden, mitigation of patient-provider tension around sensitive issues, and information communication, but also identified areas of training required, issues of security of the device, and confidentiality concerns. Patients also stated fear of forgetting how to use the tablet, making mistakes, and losing information as barriers to uptake. In pilot tests of the prototype, patients liked the feature of a recorded voice in the local language and found the screener easy to use, although many required additional help and training from the study staff to complete the screener.

**Conclusions:**

We found a self-administered digital health screener to be appealing to patients and clinicians and usable in a busy HIV clinic setting, albeit with concerns about confidentiality and training. Such a screener may be useful in improving reporting of unhealthy alcohol use for referral to interventions.

## Introduction

### Background

Alcohol consumption is a critical driver of the HIV epidemic worldwide, particularly in sub-Saharan Africa (SSA), where high-risk alcohol use and HIV are prevalent. Unhealthy alcohol use, defined as drinking more than the recommended amount of alcohol [1], is associated with increased sexual risk behavior, increased HIV transmission [2-8], and diminished treatment outcomes among people with HIV, including reduced antiretroviral (ARV) adherence [9-13] and reduced HIV viral load suppression [9,11,13-16]. Thus, reducing alcohol use among those with HIV is a public health priority.

In Uganda, more than half of the population abstains from alcohol use; however, among people who drink alcohol, most of whom are male, the yearly average consumption is 26 L of absolute alcohol, which translates to 474 L of 5.5% of alcohol by volume (typical for beer) [17]. The prevalence of heavy episodic drinking, defined as consuming at least 60 g of pure alcohol on one occasion in the previous 30 days, is 68.8% among men and 32.6% among women who consume alcohol [17]. Among people with HIV in SSA, meta-analyses have found the pooled prevalence of alcohol use disorder, defined as problem drinking that is at risk of becoming severe [18], to be 22.9% to 29.8% [19,20]. Brief interventions to reduce alcohol use have shown efficacy in reducing drinking by 15% to 30% one year after the intervention, as well as good feasibility and cost-effectiveness in primary care settings worldwide [21-28]. However, the usefulness of brief alcohol interventions depends on effective screening for unhealthy alcohol use [23,29-40], which is often underreported [41-44]. For example, we have found substantial underreporting of alcohol use by people in HIV care in Uganda to clinicians at clinic intake visits [43]. Other studies have reported high rates of underreporting of alcohol use when compared with biological measures such as phosphatidylethanol [41,44-46]. Thus, there is a need to develop methods to improve reporting of unhealthy alcohol use as an essential step toward referral to brief alcohol interventions.

### Digital Screening

The use of tablets for self-completion of clinic intake forms is increasing in many resource-rich settings; for instance, several clinical settings in the United States and Sweden have examined the use of digital screening and found that reporting of risk behaviors was comparable with more traditional screening methods [34,47-57], and the digital screening over a wide range of patient characteristics was acceptable [58-62]. Audio computer-assisted self-interviewing (ACASI) has been associated with increased reporting of stigmatized behaviors such as forced sex, especially in Africa and rural settings [63]. ACASI has also been useful for populations with low literacy, and studies have shown high levels of comfort with its use among men and women as well as in older adults across international settings [64-66]. The rise in mobile device use worldwide makes digital screening technology ubiquitous. In addition, the simplicity of touch screen computers (tablets or smartphones) allows populations with low literacy, such as in Uganda, where 24% of the adult population (aged ≥15 years) have low literacy [67], to self-administer questionnaires that would normally require the assistance of a third party. Feasible, acceptable, and efficient methods for the assessment of sensitive behaviors in settings with low literacy are essential for comprehensive HIV care. Thus, we sought to develop and test a brief (3-5 minutes), digital, easy-to-use self-administered health screener for implementation in a busy, underresourced, low-literacy HIV treatment setting.

## Methods

### Overview

We conducted patient and clinician focus group discussions (FGDs) and in-depth interviews (IDIs) to develop and pilot-test a digital self-administered screener, called the Self-Administered Digital Screener for Health (SASH), for potential use in HIV clinic waiting rooms to increase reporting of unhealthy alcohol use in people with HIV in Uganda. We examined the acceptability of the SASH by exploring what participants thought of the health screener and their experience with the SASH, including their ability to complete it on a tablet. We also explored whether the use of the SASH would be feasible in our study setting, specifically asking questions about the practical and logistical issues of implementing the new technology within standard HIV care.

### Setting

We conducted the study at the Immune Suppression Syndrome (ISS) Clinic of the Mbarara Regional Referral Hospital (MRRH) in Uganda, an 11,000-patient clinic with 45 patients seen per day per clinician. The ISS Clinic uses electronic medical records via the Open Medical Record System [68]. Adherence counseling, which sometimes includes brief advice on alcohol use, is conducted by the HIV counselors in individual and group formats at the clinic. The MRRH is a government referral hospital in the western region of Uganda with a bed capacity of 600. It is also the teaching hospital for Mbarara University of Science and Technology, home to the second-largest medical school in Uganda. The MRRH is located in the semirural city of Mbarara in southwestern Uganda, approximately 250 km from the capital city of Kampala. Although ISS Clinic patients come from throughout Western Uganda, our previous studies, from which the participants were sampled, limited inclusion to those who lived within 60 km or 2-hour driving distance of the ISS Clinic for ease of follow-up. Patient participants for this study were purposefully sampled to meet study criteria from existing research databases. All clinicians attending a weekly clinic meeting were invited to participate; during the meeting, those interested we given additional study information by study staff before providing informed consent.

The study was conducted in 4 phases summarized in Table 1, and as described in the following sections.

**Table 1:** Summary of procedures for the development of the Self-Administered Digital Screener for Health (SASH).

Goals and activities	Participants
Predevelopment phase—preliminary selection of tool options and technology: Selection of technology most suitable for the local setting by the research team Review of existing literature to inform technology options and screening content	None
Phase 1—FGDs^a^ to obtain initial input on the SASH: Obtain clinic and patient perspectives on content, format, and feasibility of use in the clinic	Pilot: male patients (n=6, mixed literacy levels) Group 2: male patients (n=7, mixed literacy levels) Group 3: clinicians (n=5, mixed sex) Group 4: female patients (n=7, mixed literacy levels) Total of 4 FGDs with 5 clinicians and 20 patient participants
Phase 2—SASH prototype development by the study team: Prototype development Translation into Runyankole and audio recording Training module development	Study team
Phase 3—iterative refinement of the SASH prototype with study participants via IDIs^b^: Obtain community, clinic, and patient perspectives on the SASH content, format, and delivery process Training and demonstration via study staff presenting parts of the intervention	First round (n=8): Male patients (n=5) Female patients (n=2) Clinician (n=1) Second round (n=7): Male patients (n=4) Female patients (n=2) Clinician (n=1) Total of 15 IDIs with 8 opinion leaders who emerged from the FGDs
Phase 4—pilot-testing of the SASH: Pilot the SASH with new patients Conduct qualitative IDIs to examine participants’ experience using the SASH	Group 1: patients with low or no literacy (9 male and 1 female) Group 2: patients who previously underreported their alcohol use (5 male and 5 female) Total of 20 IDIs with 20 patient participants

^a^FGD: focus group discussion.

^b^IDI: in-depth interview.

### Phase 1: FGDs for SASH Development, Acceptability, and Feasibility

In phase 1, we conducted 4 FGDs to elicit input on the content, format, and feasibility of the proposed screener. All FGDs were held in a private space at the clinic. Clinicians were invited to participate after the study team gave a presentation of the study during a weekly ISS Clinic meeting. We recruited patients by sampling participants from our previous study databases [69,70] (all consented to future contact) based on previous unhealthy alcohol use underreporting, sex, and literacy levels, defined as follows. Previous underreporting was defined as not meeting self-report criteria for unhealthy drinking via the Alcohol Use Disorders Identification Test-Consumption (ie, women scoring <3 and men scoring <4) [71] but having a phosphatidylethanol alcohol biomarker level of ≥50 ng/mL. Literacy (literate vs low or no literacy) was defined as the ability to read a prescribed sentence on a card when asked during study interviews. For this phase, we aimed to recruit 20 people who had underreported their alcohol use, including 10 people with low literacy and 10 people who were literate. We sought to include people with low literacy to represent the clinic population with low literacy. The FGD groups were balanced by literacy level and segregated by sex. Eligible participants were invited either via phone or in person during an Mbarara ISS Clinic visit.

We conducted clinician FGDs in English and patient FGDs in Runyankole, as desired by the participants. The discussions were conducted by a research assistant (RA; AK, hereafter referred to as the RA) while another researcher (CN) recorded the sessions, took notes, and kept time. Both are Ugandan with over a decade of HIV and alcohol research experience. For this study, the RA received training in qualitative methodology from an experienced qualitative researcher on the team (SWK) and in Dedoose (SocioCultural Research Consultants, LLC) [72] by the project director (NE). Participants were asked questions about their experience discussing alcohol use at the clinic, the acceptability and feasibility of digital screening for alcohol use, suggestions for implementing digital screening in the clinic, and suggestions for the content of a screening tool. All sessions were audio recorded, transcribed, translated into English, and uploaded to Dedoose [72] for coding by the RA. Following each FGD, the RA wrote summary notes with key observations and reflections on emergent themes, tone of the discussions, and nonverbal communication such as body language and gestures. The study team analyzed the notes using a rapid approach [73].

### Phase 2: SASH Prototype Development

We based our initial set of questions on information obtained from the phase 1 FGDs (eg, which screening questions to include) and on an existing automated screening tool using interactive voice response in a study that developed and evaluated the acceptability and use of the tool in a primary care clinic waiting room [59]. The study concluded that the use of their electronic screener in the clinic setting was feasible and accepted by both clinicians and patients, with reporting rates comparable with published written questionnaires [59]. We created a tablet-based prototype of the health screener to run on the Android operating system, the most widely used operating system in Africa [74], and used CommCare (Dimagi, Inc) [75] software as it was free and user-friendly; allowed for offline use; and had capabilities for including more than one language, pictures, and audio, which were all important criteria for implementation in a limited-resource setting. On the basis of the phase 1 FGDs, we determined that we needed to include a training module. The training module, conducted on the tablet, included written and recorded instructions on how to use the device, respond to questions, select response options, repeat questions, and end the session. Two sample questions—“What year is it?” and “How old are you?”—were included as practice questions.

### Phase 3: SASH Prototype Demonstration and Modification

We modified the SASH prototype through an iterative process that comprised 2 rounds of demonstrations, participant hands-on use, and interviews, followed by a rapid analysis and modification of the prototype’s content and appearance customization. For this phase, we selected 8 previous FGD participants, including people from each of the categories of respondents (clinicians, people with low or no literacy, and those underreporting unhealthy drinking), who emerged from the FGDs as opinion leaders to participate in the demonstration of the prototype. In each session, the RA demonstrated the SASH prototype, trained the participants on its use, and asked participants to use it in her presence. The RA solicited feedback on the wording and comprehensibility of the content in the local language, Runyankole; the layout of the screen (buttons, colors, and icons); the sound or accents of the voice recordings; and the participants’ preferences for a stylus or finger to touch the screen. The RA also requested feedback on the adequacy and effectiveness of the training module, which she summarized in written documents.

Following each individual demonstration and testing session, the RA wrote summary notes highlighting the user experience, content areas for discussion because of lack of clarity or other discrepancies, suggestions, questions, and comments raised by the participants. On the basis of a rapid analysis of the summary notes [73], we modified the prototype and conducted a second round of demonstrations with feedback solicitation 6 to 8 weeks later with the same participants.

### Phase 4: SASH Pilot-Testing and IDIs

In the final phase, we pilot-tested the SASH followed by IDIs with 20 new participants recruited from our previous studies and who met the criteria for unhealthy drinking, including 10 (50%) who had previously underreported their drinking and 10 (50%) with low or no literacy, as described previously.

The RA demonstrated the use of the SASH while the participants observed. Next, the participants proceeded to the training module, in English or Runyankole, on the tablet. Participants were allowed to use the training module repeatedly and with the RA’s (AK) assistance as needed. The RA noted where difficulties occurred during the training module and screening questions. After completion of the SASH, participants were asked to share their experiences using the SASH in a 30- to 60-minute IDI using a semistructured interview guide. All interviews were audio recorded, transcribed, translated into English, and uploaded to Dedoose by the RA for coding and analysis. Following each IDI, the RA wrote summary notes that captured the general tone of the interview and reflected on emergent themes, nonverbal communication observed, and points for discussion with the larger study team.

### Analysis of FGDs and IDIs

Two Ugandan staff members (AK and CK) and the US-based project director (NE) initially reviewed all transcripts and written summaries from all phases of the study for completeness, language, and translation accuracy. All the documents were uploaded to Dedoose for data management and coding. The 3 individuals, all experienced in qualitative analysis, worked together to code the data concurrently using both inductive and deductive methods. A predetermined set of codes was informed by the domains of inquiry explored in the FGD and IDI guides. In addition, the analysis team open-coded transcripts and summaries to identify emerging themes. Inductive codes were defined and agreed upon by the team. Next, to ensure reliability in coding, the team triple-coded the first 4 transcripts, met as a team to ensure consistency in coding, and then completed the coding. The team worked on coded data to identify themes using thematic analysis [76,77]. We used content analysis [76,77] to interpret the individual interview data, including the systematic assignment of the predetermined codes.

### Ethics Approval

The study was approved by the University of California, San Francisco Institutional Review Board (15-6933); the Mbarara University of Science and Technology Research and Ethics Committee (02/08-15); and the Ugandan National Council for Science and Technology (HS 1977). All participants provided written informed consent before study participation in their preferred language (English or Runyankole). Transportation and refreshments were provided as incentives.

## Results

### Participants

A total of 45 people (n=5, 11% clinicians and n=40, 89% patients) participated in the study. The 5 clinicians included 1 (20%) medical officer, 1 (20%) clinical officer, 1 (20%) nurse, 1 (20%) counselor, and 1 (20%) peer educator, from whom we did not collect data on age. We attempted phone contact with 114 participants from previous National Institutes of Health–funded alcohol research studies conducted by the research team at the ISS Clinic. We reached 66 people by phone, of whom 26 (39%) declined participation and 40 (61%) enrolled in the study. Participants declined mainly because of time constraints. The 40 patient participants had a median age of 38 (IQR 32-44) years, 65% (26/40) were male, 43% (17/40) were not literate, and 70% (28/40) had previously underreported their drinking (Table 2).

**Table 2:** Patient participant demographics, Self-Administered Digital Screener for Health (SASH) study, Mbarara, Uganda (N=40).

			Participants
**Sex, n (%)**			
	Female		14 (35)
	Male		26 (65)
Age at SASH interview, median (IQR)			38 (32-44)
**Literacy, n (%)**			
	Literate		23 (58)
	No or low literacy		17 (43)
**Ever underreported alcohol use (AUDIT-C^a^ negative; PEth^b^ ≥50 ng/mL), n (%)**			
	Yes		28 (70)
	No		12 (30)
**Alcohol use at last study visit, n (%)**			
	**Self-report**		
		None (AUDIT-C=0)	18 (45)
		Moderate (any self-report; AUDIT-C negative)	12 (30)
		Hazardous (AUDIT-C positive)	10 (25)
	**Self-report and PEth**		
		None (no self-report; PEth <8 ng/mL)	5 (13)
		Moderate (any self-report but AUDIT-C negative; 8≤PEth<50 ng/mL)	10 (25)
		Hazardous (AUDIT-C positive or PEth ≥50 ng/mL)	25 (63)

^a^AUDIT-C: Alcohol Use Disorders Identification Test-Consumption.

^b^PEth: phosphatidylethanol.

### Emergent Themes in Phase 1

#### Need, Usefulness, and Potential Benefits of a Health Screener

To explore the need for a health screener and the feasibility of its implementation in a busy clinic setting such as the Mbarara ISS Clinic, we conducted FGDs with clinicians and patients in which we discussed sensitive and stigmatizing health topics such as alcohol use and sexually transmitted infections. These health topics were of particular interest given the high frequency of underreporting in these areas in our previous research studies. FGD participants noted that feelings of unease between clinicians and patients were common, which made discussions of sensitive topics challenging during routine clinic visits and, therefore, discussions of this nature were often avoided in the clinic. Difficulties stemmed from both the stigmatized nature of the topics—as a patient noted, “Me, I feel ashamed to tell health workers that I drink alcohol!”*—*and the underlying stresses associated with high-volume, low-resourced clinic settings. When probed about their experiences discussing sensitive health topics, patients described disrespectful treatment and worries about time:

I fear to tell the health worker because I am worried that if I tell him this, he may insult me or respond rudely or he may decide to send me to the counselor and then I will waste all my time waiting at the counselor’s place.

Male patient, aged 28 years, literate

Clinicians unanimously described their experience with asking patients about substance use as frustrating and difficult as they felt that the patients were not truthful:

Many of them actually tell us lies if we are asking them if they drink alcohol!

Male clinician, medical officer

Encounters with patients who consume alcohol were described in the same punitive tone by all clinicians, as were their perceptions of alcohol-induced behavior, which included nonadherence to medications and risky sexual behavior among their patients who drank alcohol. Patients often feared being negatively judged if they disclosed behavior against which they had been advised by clinicians. Averting blame or punitive action was the main reason cited for not engaging in dialogue with clinicians, with whom they wanted to maintain a sense of dignity and respect:

...sometimes you go there and you really want to talk about alcohol with them, and you know, they tell us that if you are taking ARVs you should stop taking alcohol, but when you come to the clinic, knowing that you took alcohol the previous day, and you are sent to the counselor, you will not tell the counselors that you took alcohol because of the fear that they will insult you or you fear them changing their attitude towards you, so you keep quiet about alcohol or at the end of it all they will tell you to get out of their sight because they will say that for you if you decided to take alcohol instead of ARVs, there is no reason as to why they should listen to you...

Male patient, aged 37 years, low literacy

In addition, many patients felt restricted by limited time with clinicians during visits and felt that the clinicians were overworked and fatigued. Time constraints further exacerbated communication gaps and led to the prioritization of primary HIV care concerns such as medication refills. Additional delays by talking with counselors meant to some patients that “chances are high that you will go back home without medication,” for instance, in the event that the pharmacy stock ran out or the pharmacy closed; therefore, these discussions were avoided completely to ensure medication refills.

#### Privacy Concerns, Communication, and Machine Errors That Affect the Use of a Health Screener

Clinicians and patients both felt that a digital screener could aid in difficult conversations by enabling the disclosure of health issues that would otherwise be avoided in face-to-face clinic encounters. Patients expressed interest in receiving feedback on their alcohol use from the tablet itself. Patients felt that the use of a nonjudgmental device would help them articulate their issues more clearly to enable a better understanding of their problems:

I also see that it will make me happy, because the tablet will send out information that I have said, it cannot give information that I have not said. And because we never get an opportunity to explain our problems to the doctors, by the time they read my concerns from the tablet, they will know that it was really a problem.

Female patient, aged 33 years, low literacy

Younger participants (aged <40 years) with higher literacy preferred the tablet to a health care worker, particularly for discussing sensitive topics:

If I am talking to this computer, I will be comfortable, without looking at someone’s face and how they change their face whenever they are not happy with anything you did; I will not be looking at someone to be intimidated by their facial expression. Because the computer tablet asked me very well that: have you used any other medication or herbs or if you have any other sickness and then you reply yes or no. Isn’t that easy telling this to a doctor or nurse who will not listen to you but who will just be rude to you.

Male patient, aged 30 years, low literacy

Clinicians and patients both felt that a digital screener could be a useful clinic tool if they had access to reports from it before seeing patients. Facilitators to implementing such a tool in the ISS Clinic included expected decreases in patient wait times and reduced clinician workload, which would ultimately improve patient care. Clinicians felt that the use of a digital screener would also allow for more anonymity and comfort that would lead to more accurate and comprehensive disclosure:

I think they can share, no one is there, and they are not going to be penalized for the information they have given. So, I think they will be comfortable to give, to open up, they will open up about their life.

Female clinician, clinical officer

Despite the comfort levels expressed in the use of a digital device, there were concerns about loss of privacy and irreparable machine errors that could affect patient care expressed by both clinicians and patients, as well as the need to physically secure the tablet in the clinic to prevent loss:

In the event that people who keep this device lose it, a bad person may pick and then share the information with wrong people who may spread information about you.

Male patient, aged 56 years, literate

We asked the participants what they thought should be done with their answers to the screening questions. Participants’ reactions ranged from preferring that a printed paper be included in their medical records to preferring that SMS text messages or emails be sent electronically to clinicians. Concerns about electronic transmission centered on privacy and protection of personal data or unreliable network issues that would either delay or fail delivery to clinicians. Some patients thought that the device could increase communication with their providers but worried that technical problems could send the wrong information to clinicians.

#### Concerns About Time Constraints, Logistics, and Training Required to Implement a Health Screener

In response to our inquiries on the feasibility of implementing a digital screener in the clinic setting, participants expressed more practical concerns regarding time, cost, space, and effort required as prohibitive. Some participants felt that a digital screener could save time during clinic visits and ease clinician fatigue, as illustrated by an older female patient:

I think that this computer tablet is fast. You see, when you go to see a health worker then he has to ask you and this takes some time, while others are waiting to see the same doctor for the same services. But if you are using this tablet, it becomes easier and quicker compared to talking to a health worker.

Female patient, aged 69 years, literate

The aforementioned enthusiasm was tempered by clinicians’ apprehension about the additional time and effort needed for training and additional logistical challenges involved in implementing new technology on top of their heavy workload. Specifically, clinicians worried about added time, staffing, and clinic flow disruptions that would result from added training in a clinic setting that was already understaffed with limited resources. This concern was also voiced by patients who worried about their ability to understand and operate a novel digital device.

### Phase 2

The first version of the SASH prototype was developed by the study team (phase 2). We considered 5 topics (pain, smoking, alcohol use, physical activity, and depressed mood) used in a previous waiting room screener [59] plus ARV adherence. The chosen topics for inclusion in the SASH were agreed upon by the study team and were based on what we considered to be the most relevant in our study setting from our previous experience in the Mbarara ISS Clinic. It was also important for the study team to screen for behaviors that would allow for feasible interventions in the future in this setting. We included questions about ARV adherence in the SASH as it was frequently discussed during the FGDs and we felt that including general health questions along with questions on sensitive behaviors would help normalize the SASH as a general clinic tool, making it more acceptable to patients in this setting where sensitive behaviors such as alcohol use are often stigmatized. We used validated questions on adherence [78], depression [79], and alcohol use [71].

**Figure 1:**
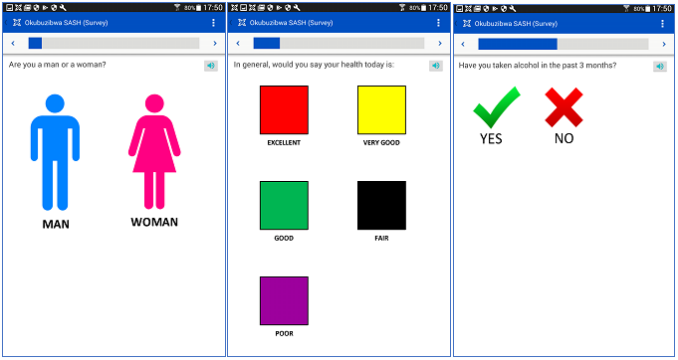
Sample Self-Administered Digital Screener for Health (SASH) questions with color-coded response options.

The final content of the SASH included a training module with 2 questions plus 13 questions that covered sex, general health status, adherence to ARVs and other medicines and herbs, alcohol use, sexual health, and depression, as well as a final question asking participants if they wanted to talk to a clinician about their health issues (Table 3). The responses to this last question, as with others in the SASH, were not followed by clinic referrals as the scope of the study was limited to testing the SASH. The SASH included color-coded multiple-choice questions and response options (Figure 1), with an additional option for participants to play the corresponding audio clip by pressing the audio symbol shown. The entire SASH was available in English or Runyankole, the local language.

**Table 3:** Self-Administered Digital Screener for Health (SASH) training module and screener topics, questions, and response options.

SASH topics and sections		SASH instructions and questions	SASH response options
**SASH training module**			
	Introduction	Hello! We are testing out this small computer for possible use in the clinic. First, we want to show you how to use this small computer. If you want, you can listen to someone read each question out loud. To hear a question, click on the picture of the speaker on the right. Whenever you see the picture of the speaker, you can touch it to hear the question read out loud. You do not have to touch it very hard, but do give it a little tap. Tap it again if you want to stop the voice. Please tap on the speaker picture now to hear a message.	N/A^a^
	Example 1a	This small computer will ask you questions. The answers to these questions will sometimes have a picture or a color connected to them. The small computer will not take your picture. To answer the question, you will tap on the picture or color of the choice that you feel answers the question the best. Please tap the arrow to try an example question.	N/A
	Example 1b	What year is it?	1997 (red) 2007 (black) 2017 (yellow)
	Example 2a	Great! To type numbers into the small computer, you will tap the numbers that you will see at the bottom of the screen. When you are done tapping the numbers, you will need to tap the arrow to move to the next page. Please tap the arrow to try an example question.	N/A
	Example 2b	How old are you?	N/A
	Example 3a	Good! Please tap on the green bar above to finish the training.	N/A
**SASH screener**			
	Introduction	Hello! Please answer each question as best you can.	N/A
	Sex	Are you a man or a woman?	Man (image) Woman (image)
	Health	In general, would you say your health is today:	Excellent (red) Very good (yellow) Good (green) Fair (black) Poor (purple)
	HIV medication adherence	Have you had any trouble taking your ARVs^b^ lately?	Yes (green check) No (red X)
	HIV medication adherence	How have you been at taking your ARVs in the last 4 weeks?	Excellent (red) Very good (yellow) Good (green) Fair (black) Poor (blue) Very poor (purple)
	Other medications	Are you currently taking any herbs or medicines other than those you are given here at the clinic?	Yes (green check) No (red X)
	Alcohol use	Have you taken any alcohol in the past 3 months?	Yes (green check) No (red X)
	Alcohol use	On how many days have you had at least one drink of alcohol in the last 4 weeks?	3 or more days per week (green) 1-2 days per week (yellow) 2-3 times in the past 4 weeks (purple) 1 time in the past 4 weeks (red) Never in the past 4 weeks (blue)
	Alcohol use	How often did you have 6 or more drinks of alcohol on one occasion in the past 3 months?	Never (red) Monthly (blue) Weekly (black) Daily or almost daily (yellow)
	PHQ^c^	Over the past 2 weeks, how often have you been bothered by having little or no interest in doing things?	Not at all (red) Several days (black) More than half the days (purple) Nearly every day (yellow)
	PHQ	Over the past 2 weeks, how often have you been bothered by feeling down, depressed, or hopeless?	Not at all (red) Several days (black) More than half the days (purple) Nearly every day (yellow)
	STIs^d^	Have you had any symptoms of a sexually transmitted infection recently?	Yes (green check) No (red X) Don’t know (black question mark)
	Other discussion with health worker	Is there anything else you might want to talk to a health worker about?	Yes (green check) Check No (red X)
	Other discussion with health worker	Thank you for answering all of these questions! Do you want to talk to a health worker about these things today?	Yes (green check) Check No (red X)
	Other discussion with health worker	Would you like us to text a health worker or print out your information on a piece of paper for you to give them?	Print (yellow) Text (red) Both (purple) No preference (black)
	Final thank you	Thank you! You have answered all of the questions. Please tap on the green bar above to finish the screener.	N/A

^a^N/A: not applicable; response not required.

^b^ARV: antiretroviral.

^c^PHQ: Patient Health Questionnaire Depression Scale.

^d^STI: sexually transmitted infection.

### Emergent Themes in Phase 3: Additional Training and Modifications to Screener Questions

The SASH was further refined through an iterative process with the patients (phase 3). Following the demonstrations of the SASH, patients provided input on the content, order of questions, and mode of delivery. We determined that we needed to allow for multiple training sessions after all participants requested additional training time and repeated the training module several times. Thus, we separated the training module from the actual screening questions to allow participants to repeat the training module until they felt comfortable with and confident about the technology. The inclusion of multiple-choice as opposed to open-ended response options, particularly with questions on the “number of days drinking,” was also guided by feedback from patients who felt that it was difficult to recall these measures with accuracy. Both clinicians and patients in the FGDs felt that it was best to place perceived difficult questions, such as those about depression, at the end. Some suggestions were outside the scope of a screening tool (eg, a female patient suggested that the digital screener serve as a medication adherence intervention).

### Emergent Themes in Phase 4

#### Time and Comprehension Needed to Complete the SASH

In this phase, it took 20 participants approximately 20 minutes to complete the training module and 30 minutes to complete the screener. The RA noted that a few participants took >1 hour to complete the screener as they requested additional training sessions and demonstrations with the RA. When given the option to use audio or read the screener, the RA noted that more than half of the participants (14/20, 70%) chose the audio option, including 15% (3/20) who were literate.

#### General Satisfaction, Feelings of Empowerment, and Self-reflection With the SASH

Participants testing the prototype in phase 4 reacted positively to the SASH. They liked it, found it easy to use, and found the local language (Runyankole) option appealing, as expressed by one of them:

What was so easy is the fact that I could use it in my language, Runyankole.

Male patient, aged 41 years, literate

Participants appreciated the short length of the screener, simplicity of the screening questions and response options, and clarity of the instructions. A female participant with low literacy explained that “it tells and guides you on what to do and that’s what I liked,” and others found this particularly helpful in building confidence to proceed from the training module to the screening questions with ease:

I have liked it because it has short questions and they explain to the point. You read the question and you quickly understand it and you find a way of answering it. And each question has its own answer.

Male patient, aged 56 years, literate

The availability of multiple choices for responses was empowering to some:

The instructions made me happy because they gave me an option if I did not like something, then I would choose something else altogether. It shows different responses for example if one is weak or strong, sick or not sick, so one chooses accordingly. It shows what you are, it does not disagree with you but lets you choose.

Female patient, aged 39 years, literate

The audiovisuals and colorful images simplified the experience and eased comfort levels, especially for participants with low literacy and limited experience with technology:

They were good, the fact that there are pictures, if one knew how to read, all would be fine. But for some of us that don’t know how to read, you can’t comprehend, you just see the pictures and try to follow...

Female patient, aged 43 years, low literacy

A male participant with low literacy likened the SASH to his smartphone: “I did not fear because I use a smart phone every day.” Another female participant with low literacy commented on how accomplished she felt in using such novel technology:

I felt good, when I saw that it is a touch screen, I wondered, is this the technology that I have been hearing about. I then said to myself, let me see it. When I get home, I will narrate to people I used technology that I have never used before.

Female patient, aged 43 years, low literacy

In some cases, answering questions prompted patients to self-reflect on their health and behavior. In addition to self-reflection, some participants felt that the SASH held them accountable and compelled honesty in their communication about their health with their providers:

Devices that keep secrets like this one...if an expert opens it and goes through it, he will not fail to get something. So better you speak the truth so that in future, it does not reveal your lies or report you.

Male patient, aged 49 years, literate

#### Training Concerns and Suggestions for Improvement of the SASH

Many patients were concerned about making mistakes in their responses or forgetting instructions while completing the SASH. We noticed this when demonstrating the SASH to participants and also during the training module. However, all participants, including those with low literacy, were open and eager to use the technology with adequate instruction and guidance. Participants felt that multiple training sessions and ongoing instruction would facilitate the implementation of the SASH:

...but it’s important to come back and repeat the training. Even those that study cannot study just once and finish.

Male patient, aged 44 years, low literacy

Although it took participants much longer than expected to complete the SASH (approximately 1 hour vs 3-5 minutes), particularly for those who took over an hour, it seemed that the time spent on the SASH was less of a concern to participants than their concerns about being accurate and thorough. Some participants expressed worries about forgetting how to use the tablet and making mistakes and feared the loss of information and damage to the device. Only a small number of participants voiced the need for improvements. In all, 10% (2/20) of female participants with low literacy suggested adding a video to enable a more personable experience with the nonhuman device.

Participants had varied understandings of the functions of the SASH. An ancillary finding during analysis was that our translation of “digital screener” was not precise because of the limited vocabulary in the local language for a specific description. Therefore, “digital screener” was translated to a more general term such as “technology” in Runyankole. Some participants clearly understood the SASH as a screener, whereas others had broader expectations of its function and ability to provide health care. The latter group sometimes viewed the SASH as an educational tool, a digital suggestion box for lodging complaints, a tool to intervene on alcohol use and other behavioral issues, or even as a complete replacement for health care providers:

It taught me to completely stop alcohol and then I will have good health. Now if you go by the advice given by this technology, it educates you and if you go by the rules, you become healthy and you live a longer life.

Male patient, aged 52 years, literate

After the patients used the SASH, we asked them about their preference for alcohol use screening with the SASH versus a clinician. Participants reported a range of responses, from the preference for SASH to clinician preference. Those who preferred the SASH expected that it would spare them the discomfort of dealing with difficult encounters with clinicians as well as maximize their time at the clinic:

It is better than the provider because the provider will get out of her mood and then he shouts at you but as for the technology, it will never get out of its mood. You input what you want and then the provider will read it. The technology doesn’t act in a mean way.

Male patient, aged 41 years, literate

The participants who preferred in-person interactions with clinicians felt that the SASH lacked flexibility in responding to issues not already programmed or in responding at all. This was coupled with concerns about grasping the technology:

You see I do not see the person talking in the tablet, yet for the clinician we are face to face. For example, If I had a wound, the tablet would not be able to see it or prescribe medication for me, yet the clinician can do it. The tablet cannot bandage my hand.

Female patient, aged 42 years, low literacy

For some, the preference for a digital tool versus direct contact with a clinician was clearly not an issue as they expressed the expectation of similar outcomes for both:

There is no difference between the two. When the clinician asks you, you answer him and when the device asks you, you have to give an answer. So, no difference.

Female patient, aged 56 years, low literacy

## Discussion

### Principal Findings

We developed and pilot-tested a touch screen, digital health screener with the potential to increase reporting of unhealthy alcohol use in people with HIV in Uganda, a low-resource setting with varying levels of literacy and reasons for underreporting alcohol use. The resulting SASH is a health screener with 15 screening questions illustrated with colorful images and a voice option that reads the questions and response options to the participants. We found that the SASH was acceptable to clinicians and patients who consumed unhealthy levels of alcohol, including those with a history of underreporting their alcohol use, and usable by patients with a range of literacy levels. Although our scope did not include feasibility studies, we explored patient and clinician perceptions of the feasibility of implementing a digital screener in their clinic setting. Their responses focused on practical concerns such as where and when to use the tablet within the clinic; charging the tablet; cost; time taken to complete the screener, including training; and the potential additional workload for clinic staff who would have to train patients to use the tablet and screener. Clinicians and patients in our study shared a desire for the SASH to be used to improve patient care through data sharing with clinicians to mitigate communication barriers between patients and clinicians and save clinicians’ time.

Training, literacy, and privacy of information were the primary concerns regarding the use of a digital screener in this low-resource and low-literacy setting. We found that including a training module preceding the screening questions was crucial. The fear of “failing” the screening or mishandling the device made participants particularly anxious, and all (20/20, 100%) requested retraining with the RA during testing because of this. It was unclear whether these concerns about breaking the device while handling it were rooted in patients’ general concerns about the punitive consequences of making mistakes or mere fear of using new technology. Nonetheless, a notable finding in all phases of this study was that additional training beyond the basic instructions and demonstration of the prototype, including repeated practice sessions, was critical for many clinicians and patients. Training was emphasized as essential regardless of the participants’ previous experience with smartphones, electronic devices, or other technology. The need for additional training, as described previously, was similarly noted in another study of new technologies in a clinic setting in SSA [80], as well as in previously mentioned studies that tested ACASI in settings with older adults and patients with low literacy [64-66].

### Comparison With Prior Work

Our results are consistent with previous studies that demonstrated the feasibility and acceptability of digital and web-based previsit screening for substance use [59,81-87]. Although most of these studies were conducted in high-income countries, the results of those conducted in SSA are promising as well [88-92]. A meta-analysis has shown that digital screening followed by brief alcohol interventions is effective in reducing weekly drinking [93]; however, few studies have focused on whether digital screening increases entry into interventions. By contrast, this study aimed to develop a screening tool as a critical precursor for intervention in a setting where alcohol use is both prevalent and stigmatized [43].

We found evidence of tensions between health care providers and patients that affected their ability to discuss sensitive health topics, including sexual health and substance use, during routine clinic visits. This is consistent with a qualitative study with patients and providers in a rural primary care clinic in the United States that found patient-provider relationships to be critical in the feasibility of substance use screening and that patients’ preference was for self-administered, tablet-based screeners [94]. Clinicians in our study expected patients to underreport their drinking but also expressed frustration when patients were not forthcoming about their drinking on consultation. Similarly, patients frequently described their interactions with providers as hostile, with punitive consequences for behaviors considered to be unacceptable, including unhealthy alcohol use. Therefore, avoidance of punitive consequences was a reason for underreporting in addition to maintaining dignity in the presence of health care providers. The preservation of social status for people with HIV, already burdened by the stigma of HIV, was noted as a reason for socially desirable reporting in this study. Both clinicians and patients felt that clinicians did not have enough time for patients during clinic visits and, as such, deeper explorations of mental health and behavioral issues in the context of current HIV care were limited. These findings suggest that digital screening methods may help mitigate the barriers imposed by face-to-face questioning on sensitive topics. More private and anonymous screening methods may prove particularly useful in settings where sensitive behaviors are stigmatized and pressures within patient-provider relationships prohibit accurate self-reporting.

Our success in developing and pilot-testing the SASH with 20 people with HIV who drank alcohol and had varied levels of literacy in the Mbarara ISS Clinic could potentially be replicated and scaled up in similar settings. Although this screener primarily focused on alcohol use, waiting room screening tools have the potential to efficiently and sensitively screen for key health issues such as mental health and medication adherence and ultimately lead to improved referral and treatment for several health and psychosocial issues.

### Limitations

This study had some limitations. The time required to train patients to use a new digital device may have diminished the focus on its actual use as a screener and may have negatively influenced perceptions. We intended for the SASH to be brief (3-5 minutes), but it took participants a relatively long time (approximately 1 hour or more) to complete the 15-question screener with a training module. Furthermore, our translation of “digital screener” more generally as “technology” in Runyankole may have affected patients’ understanding of the larger context of the SASH as a screener and limited their input on content development as well as their discussion of its specific use as a screener in the clinic. Therefore, feedback from some patients focused on the use of the SASH as a learning tool, medical resource, or digital suggestion box to which complaints about their care could be lodged rather than on its use solely as a screener. It was beyond the scope of this study to provide the SASH results to clinicians and determine their impact on patient care or clinical practice. Finally, given our limited scope, we could not fully explore the feasibility of implementing the SASH in this clinic setting.

### Conclusions

Our findings suggest that a digital health screener has the potential to improve reporting of unhealthy alcohol use in clinical care for referral to alcohol interventions in HIV clinics in low-resource settings. Further studies are needed to determine the efficacy of the SASH in improving self-reporting and further develop means of implementation.

## Abbreviations

ACASI: audio computer-assisted self-interviewing
ARV: antiretroviral
FGD: focus group discussion
IDI: in-depth interview
ISS: Immune Suppression Syndrome
MRRH: Mbarara Regional Referral Hospital
RA: research assistant
SASH: Self-Administered Digital Screener for Health
SSA: sub-Saharan Africa
